# PM2.5 exposure induces vascular dysfunction via NO generated by iNOS in lung of ApoE-/- mouse

**DOI:** 10.7150/ijbs.36073

**Published:** 2020-01-01

**Authors:** Min-Hui Long, Xiao-Ming Zhu, Qin Wang, Yao Chen, Xiang-Dong Gan, Fei Li, Wen-Liang Fu, Wei-Wei Xing, Dong-Qun Xu, Dong-Gang Xu

**Affiliations:** 1 Institute of Military Cognitive and Brain Sciences, Beijing, 100850, China; 2 National Institute of Environmental Health Chinese Center for Disease Control and Prevention, Beijing, 100021, China

**Keywords:** PM2.5 exposure, iNOS, Oxidative stress, Inflammation, Vascular function

## Abstract

PM2.5 exposure exacerbates cardiovascular diseases via oxidative stress and inflammation, the detailed mechanism of which is unclear. In this study, the effects of oxidative stress and inflammation, as well as vascular structure and function were studied by multiple PM2.5 exposure model of ApoE-/- mice. The results indicated that NO produced by iNOS not cNOS might play important roles in inducing vascular dysfunction after PM2.5 exposure. The occurrence order and causality among NO, other oxidative stress indicators and inflammation is explored by single PM2.5 exposure. The results showed that NO generated by iNOS occurred earlier than that of other oxidative stress indicators, which was followed by the increased inflammation. Inhibition of NOS could effectively block the raise of NO, oxidative stress and inflammation after PM2.5 exposure. All in all, we firstly confirmed that NO was the initiation factor of PM2.5 exposure-induced oxidative stress, which led to inflammation and the following vascular dysfunction.

## Introduction

With the development of industry and the acceleration of urbanization process, air pollution has become a common risk for human health, especially in developing countries 1-3. The atmospheric fine particulate matter (particulates of aerodynamic diameter less than or equal to 2.5 microns suspended in the air, PM2.5), as a major kind of air pollution, is one of the main factors causing diseases, especially the respiratory and cardiovascular diseases 4-8. It has been also declared as an etiological factor of cardiovascular disease by the American Heart Association in 2014.

The inflammation, oxidative stress and vasculopathy are three key factors involved in the development of cardiovascular diseases, all of which are closely related to each other 9-11. Oxidative stress is a common inducer of chronic inflammation. In addition, ROS can induce the haemoendothelial cell to generate and secrete MMPs, ECM and so on, which are involved in reconstruction of the extracellular matrix 12-14. The PM2.5 composition is complex, which is mainly composed of a variety of organic matter, inorganic salt ions and some transition metals, some of which can induce oxidative stress and inflammation, as well as vasculopathy. Lung is the direct target organ of PM2.5 exposure. The local inflammation and oxidative stress in lung induced by PM2.5 exposure may affect cardiovascular system through diffusion in blood circulation, and finally lead to the increased vasculopathy, as well as the deterioration of cardiovascular diseases.

NO (Nitric oxide, NO) is an important signal molecule involved in various physiological and pathological processes including oxidative stress, inflammatory reaction and vasodilatation. Nitric oxide synthase (NOS) catalyzes the production of NO from L-arginine. There are three isoforms of NOS: neuronal NOS (nNOS), endothelial NOS (eNOS) and inducible NOS (iNOS), which have differences in their distribution, regulation and the ability to produce NO 15-20. Both nNOS and eNOS belong to constitutive NOS (cNOS). Under normal physiological conditions, a small amount of NO is synthesized by cNOS to maintain the body's normal physiological activities, including the regulation of vasodilatation, anti-inflammation and antioxidation 21, and iNOS is almost not involved in NO synthesis. While in pathologic state, the expression of iNOS is abnormally increased in stress response, and NO causing body oxidative damage is mainly synthesized by iNOS 22. Under this condition, NO at an abnormally high concentration can react with oxygen or peroxide to generate nitrogen dioxides, then and lead to oxidative stress. In addition, the excessive NO also contributes to the proliferation of inflammatory cells and the release of inflammatory cytokines, accelerates the tissue damage through oxidative stress and inflammation, strengthens vasoconstriction, and then results in cardiovascular disease 23-25. More than this, vascular contraction and relaxation also can be regulated by NO, which maintains the function of blood vessel and keeps blood pressure stable. It has been reached a consensus that NO, inflammation, oxidative stress are all involved in the toxicity of PM2.5 exposure, of which the detailed mechanism to induce vascular dysfunction and exacerbate the cardiovascular diseases is still unclear. In this study, ApoE-/- mice exposed to PM2.5 (sampled from Beijing city in the winter) were used to detect the dynamic changes of NO, oxidative stress and inflammation, and to evaluate the alteration on the blood vessel, so as to explore the underlying mechanism of vascular dysfunction caused by PM2.5 exposure.

## Materials and methods

### Collection and preparation of PM2.5 samples

A sampler with the middle flow rate of 77.59 L/min (model: TSP/PM10/PM2.5, Beijing Geological Instrument-Dickel Co,Ltd.) was used to collect PM2.5 onto filter membrane (Whatman® 41 filters, Whatman Inc, Maidstone, UK) at the altitude of 40 meters between the 2nd and 3rd ring road from Oct. to Dec. 2015 in Beijing China.

### Chemical analysis of PM2.5 samples

Plasma emission spectroscopy was utilized to determine the inorganic components (ICP-AES, Model: ULTIMA, JOBIN-YVON Company, France). PAHs were analyzed by HPLC (Water® 2690 Separations Module, Water® 474 Scanning Fluorescence Detector and Symmetry® C18 3.9 mm×150 mm, 65μm column). The other organic components were analyzed by high performance liquid chromatography combined with mass spectrometry.

### Animal models

120 male ApoE-/- mice (genetic background:C57BL/6 ) aged 8 weeks old were purchased from Peking University Medical Science Center, which were fed on in the 2nd grade of animal feeding room at constant temperature (20±2℃) and humidity (relative humidity60%), and were adaptively fed for one week, then fed on high fat diet (containing 2% cholesterol and 10% fat) for 20 weeks. This study was carried out in accordance with the Regulations of Experimental Animal Administration issued by the Ministry of Science and Technology of the People's Republic of China. All animal protocols were approved by The Animal Ethics Committee of Institute of Military Cognitive and Brain Sciences.

### Instillation to PM2.5

40 ApoE-/- mice aged 29 weeks old were anesthetized by 0.08% pentobarbital sodium (40mg/kg) and were instilled with PM2.5 at dose of 4mg/kg body weight as PM2.5 exposure group. 40 mice were instilled with normal saline at the same volume as control, and 40 mice were instilled with PM2.5 with the same dose and received intraperitoneal injection of L-NAME as L-NAME group, 10 mice weren't treated as blank group. 6 mice were randomly taken, respectively from the PM2.5, inhibition and control group at 0, 0.5, 1, 6, 24hrs after the first instillation for blood sampling, pulmonary lavage and lung tissue. The remaining mice from PM2.5 group continued to be exposed to PM2.5 once every two days for another 2 times. Control group also continued to be instilled with 0.1mL saline every two days, the blank group mice received no treatment. 24hr after the last instillation, all the mice were anesthetized by 0.08% pentobarbital sodium (40mg/kg) via ascending aorta, and the blood was sampled from the abdominal aorta with 2mL anticoagulant vial (Beijing Ji Shui Chuang Ge Research Institute of Medical Instruments, Beijing), then blood plasma was separated and collected by centrifugation for 10min at 3000r/min and 4℃.

### Vascular physiology assay

The relaxation and contraction experiments of ascending thoracic aorta were conducted in accordance with literature's method 26.

### Detection of biochemical indexes

The levels of ET-1, IL-6 and TNF-α in serum, lung or bronchoalveolar lavage fluid were tested (Abcam Company, America). ELISA was performed to detect SOD. according to the instructions provided by Bei Jing Zhi Li Sheng Ke Technology, which was also detected by Hitachi 7100 Fully Automatic Biochemical Analyzer. ROS was determined by flow cytometry with ROS kit purchased from Bei Jing Zhi Li Sheng Ke Technology Co. Ltd. iNOS, eNOS and nNOS were detected with ELISA kit provided from Bei Jing Zhi Li Sheng Ke Technology Co. Ltd. NO detection was carried out following the instruction (BI YUN TIAN company). AIIiance HPLC system (Waters, American) was used to test BH4 (Sigma, American). MDA was tested with TAB means according to protocol (Nanjing Jiancheng biotech company, China). All the detections were completed by the same technologist.

### Pathological examination

The mouse aortic arch and lung were taken and cut into 0.5cm^3^ for hematoxilin-eosin (HE) 27. The morphological change and inflammatory infiltration were observed by optical microscopy, and the pathological conclusion was made by two pathologists.

### Immunohistochemistry

Aortic arches and lungs were embedded in paraffin for detection by IHC. Immunostaining was performed using antibody against iNOS, eNOS andnNOS respectively. Biotinylated anti-rabbit IgG (Santa Cruz, USA) was used as the secondary antibody, and the nitrotyrosine staining was visualized using Vectastain ABC kit and DAB as the substrate (Vector Laboratories, USA).

### RNA and DNA extraction and real-time quantitative PCR assay

The total RNA was extracted (Biomed, Primer Script Reverse, China) and cDNA was synthesized with TaKaRa Kit (TaKaRa Biotechnology Co Ltd, Jan), DNA extraction Kit (Beijingzhili Biotech) was adopted to prepare the DNA for iNOS methylation detection, which was carried out with Kang Wei methylation kit (Beijing Kang Wei co Ltd., Beijing) according to its manual. The Primers for methylation detection were listed in Table 1. The qPCR was performed according to the instructions. The cDNAs were used for real-time quantitative PCR with MaximaTMSYBR Green/ROX qPCR Master Mix (2×) in Mx3000P qPCR Systems (Agilent Technologies Stratagene Products Division, USA) to detect IL-6, TNF-α, MMP9, TIMP1, eNOS, iNOS and nNOS , and all primers for qPCR were shown in Table 2.

### Western blotting

RIPA lysis buffer and Lowry assay kit were provided by Bei Jing Zhi Li Sheng Ke Technology Co Ltd. The antibodies against TNF-α, IL-6, iNOS, eNOS, nNOS and ET-1 were bought from Abcam Co Ltd., HRP-labeled 2nd antibodies and the house-keeping gene GAPDH were supplied by EMB Biotechnologies Co. Ltd.

### Statistical analysis

All parameters were expressed as mean±SD. The variant values of blood vessel function were evaluated by P value, p<0.05 indicating a significant statistical difference, p<0.01 indicating a very significant statistical difference. Post hoc Bonferroni correction was used to perform multiple analyses. The differences of the results between immunohistochemistry and RT-PCR were analyzed by pairing t test. All the statistical analyses should be done by the software SPSS13.0 (GraphPad Software, Inc., San Diego, CA).

## Results

### The influence on inflammation, oxidative stress and the vascular structure after multiple exposures to PM2.5

**Figure 1:**
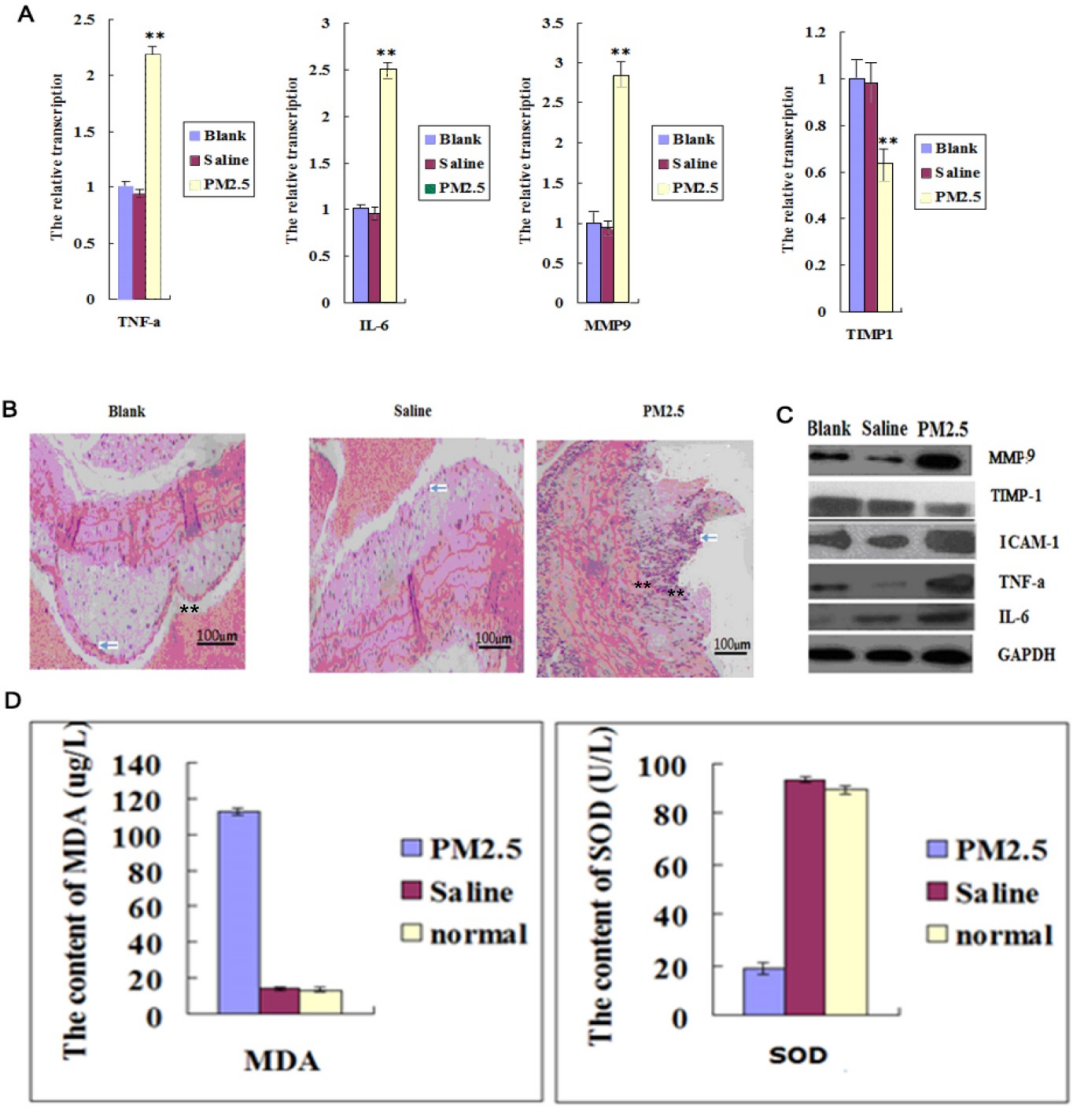
**The effects of multiple exposures to PM2.5 on the vascular structure, inflammation and oxidative stress. (A)**The qPCR assay showed the transcription levels of inflammatory factors in vessel (n=5). **(B)** The representative HE results showed the infiltration of monocyte in vessel and plaque (n=3). **(C)** Western blot analysis for the protein levels of inflammatory factors in vessel (n=3). **(D)**The oxidation stress level was detected in vessel (n=5). **P<0.01 compared with the blank group.

The intravascular levels of IL-6, TNF-α and ICAM-1 were significantly increased after exposure to PM2.5 for three times (Fig. 1A and C). In addition, a large number of inflammatory cells infiltrated into the mouse blood vessel, indicating that vascular permeability was increased (Fig. 1B). TIMP-1 (tissue inhibitors of metalloproteinase-1, TIMP-1) was significantly decreased, while MMP9 (Matrix metallopeptidase 9, MMP9) in vessel was increased greatly (Fig. 1A and C), suggesting an altered vascular structure after PM2.5 exposure. In addition, exposure to PM2.5 significantly decreased the SOD activity in blood while remarkably increased MDA level, indicating an enhanced oxidative stress caused by PM2.5 exposure (Fig. 1D).

### The multiple exposures to PM2.5 activated iNOS, but not cNOS

**Figure 2:**
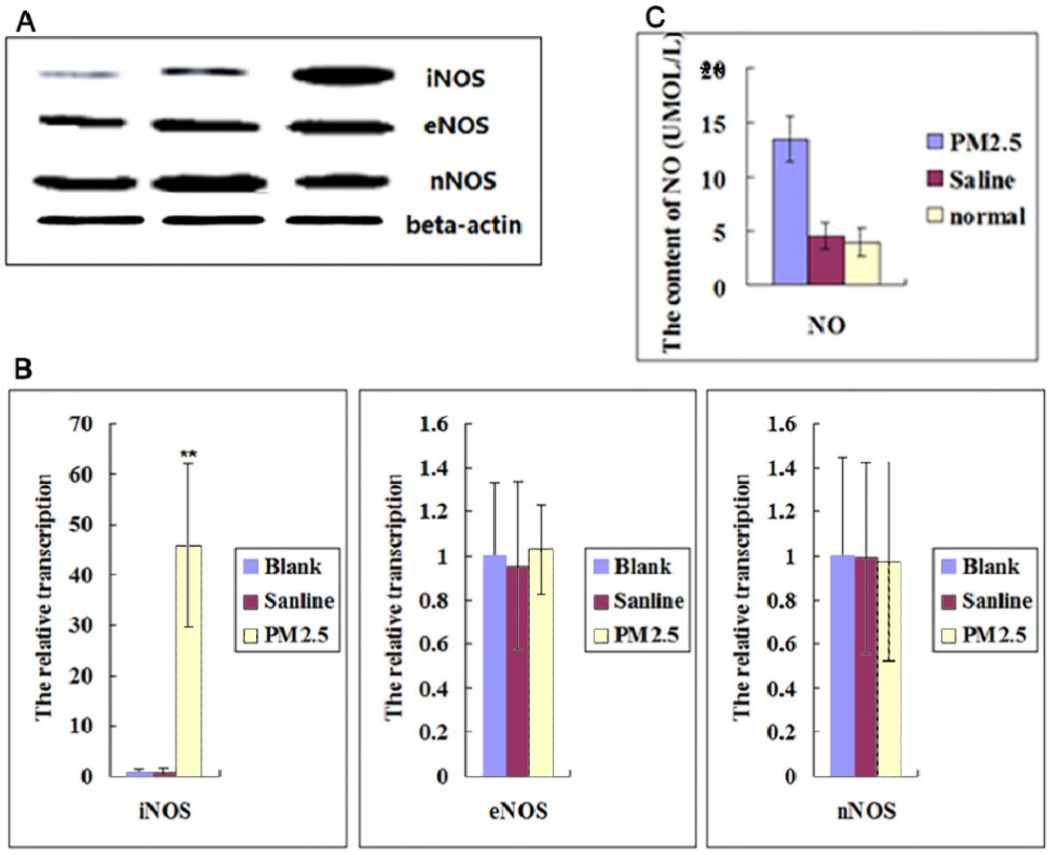
**The effects of multiple PM2.5 exposure on the NOSs in vessel and NO in blood. (A)** Western blot analysis for the protein levels of NOSs in vessel (n=3). **(B)**The qPCR assay showed the relative transcription levels of NOSs in vessel (n=3). (C)NO level was detected in blood (n=5). **P<0.01 compared with the blank group.

NO is an important ROS inducer, which is involved in inflammation. So we further checked the NOSs expression in Blood Vessel. The results showed that neither the expression level of eNOS nor nNOS in vessel was significantly changed after multiple exposures to PM2.5, but the expression of iNOS was significantly increased (Fig. 2A, B).It was also found that NO levels in blood were upregulated significantly (Fig. 2C). These results suggested that iNOS-induced NO played important roles in the effect of exposure to PM2.5.

### Alterations in vascular tone induced by multiple PM2.5 exposure

**Figure 3:**
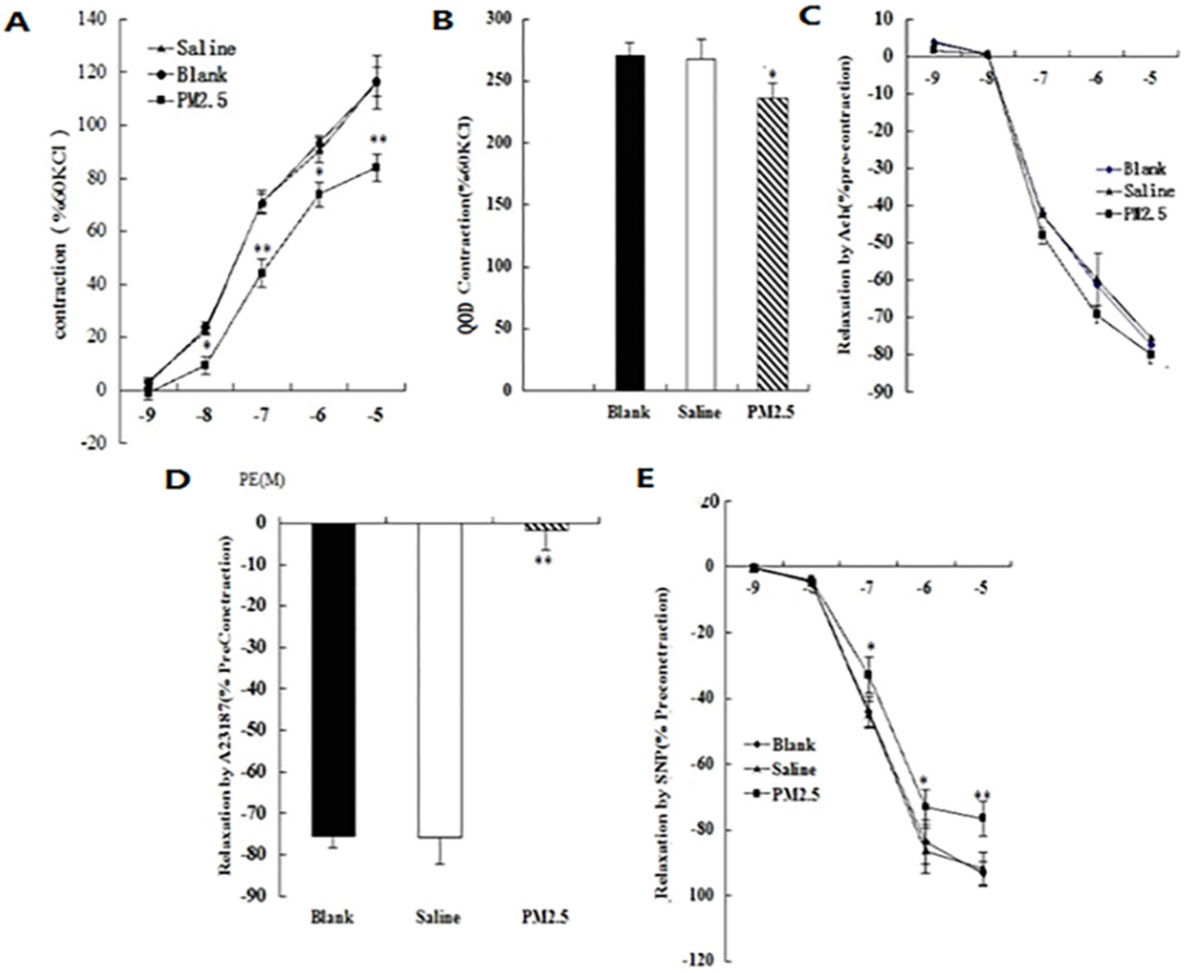
The vascular tone after PM2.5 exposure. (A) The systolic responses of the aortic ring after Phenylephrine (PE) stimulation (n=5). (B) The effect of ODQ treatment on the systolic responses of the aortic ring after PE stimulation (n=5). (C) The response values of the aortic ring after Ach stimulation (n=5). (D)The diastole of the aortic ring after Ca^2+^ ionophore A23187 treatment (n=5). (E)The vessel diastole after sodium nitroprusside (SNP) treatment (n=5). *P<0.05 compared with the blank group, **P<0.01 compared with the blank group.

In addition to oxidation stress and inflammation, NO is also closely related to vasodilatation. To test the changes of blood vessel tension after exposure to PM2.5, the intact aortic ring from different groups were prepared. When stimulated with KCl at the concentration of 60 mmol/L, the value of the systolic responses for the aortic ring in normal saline control, blank control, and PM2.5-exposed animal were 2.42 ± 0.17 g, 2.44 ±0.21 g, and 2.38 ±0.35 g (p > 0.05), respectively. The systolic responses for the aortic ring in the three groups were significantly increased after Phenylephrine (PE) stimulation, and positively correlated with its concentration. After stimulation with PE at a concentration of 10^-5^, the response values in the normal saline, blank control and PM2.5 group were increased by 135 ± 7%, 136.4 ± 8.3%, and 82.1 ± 5.4% respectively (Fig. 3A). However, the differences of sensitivity of the aortic ring to PE (50% of the maximum response value) were not statistically significant among the three groups (CI: 0.06-0.25, 0.05-0.24, and 0.07-0.26, respectively). In order to detect the effect of the NO/sGC signal on the PE-induced systolic function of the aortic ring, the sGC inhibitor ODQ was added when PE reached to the maximum response concentration at 60 mmol/L KCL. It was found that the PM2.5-induced decrease of the aortic ring intension completely recovered after ODQ treatment, illustrating that the abnormality of the NO/sGC signal occurred when exposed to PM2.5 (Fig. 3B). The response values of the aortic ring to Ach stimulation in the three groups were not statistically different (Fig. 3C), but Ca^2+^ ionophore A23187 significantly reduced the diastole of the aortic ring in the PM2.5 groups (P<0.05) (Fig. 3D). PM2.5 could significantly eliminate the vessel diastole caused by sodium nitroprusside (SNP) (Fig. 3E) (93.55±3.76, 94.11±2.98, 81.3±2.22 for the three groups, respectively.). The results suggested that exposure to PM2.5 may influence the release of NO to enhance vasoconstriction. In sum, our data strongly suggested that NO played important roles in the pathologic effect caused by PM2.5 exposure.

### The dynamic changes of NO in the lung, blood and vessel after single exposure to PM2.5

**Figure 4:**
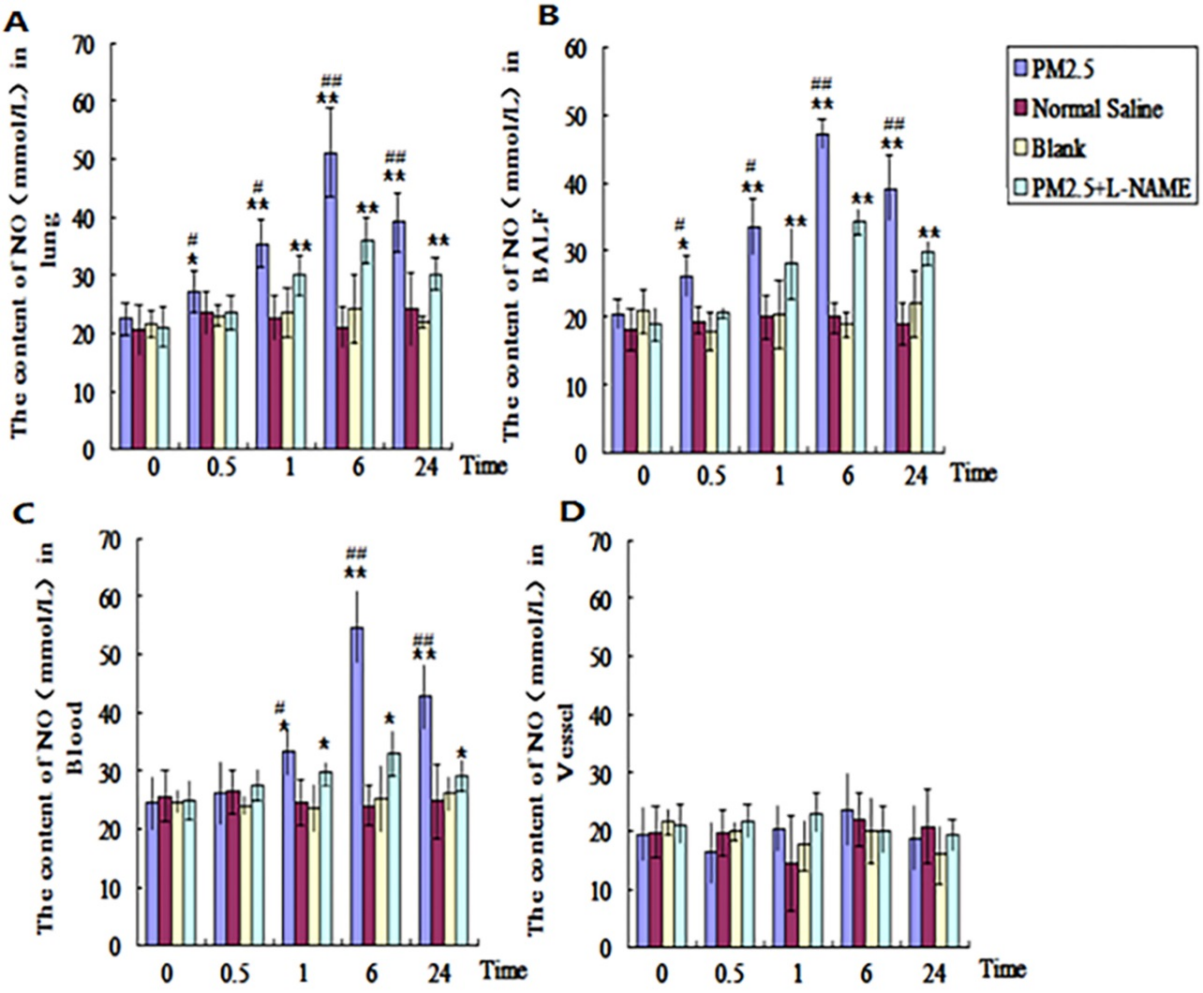
**The dynamic effects of single exposure to PM2.5 on NO level in different tissues. The NO level was detected in different tissues. (A)** Lung (n=10). **(B)** Bronchoalveolar lavage fluid (BALF) (n=10). **(C)** Blood (n=10). **(D)** Vessel (n=10). *P<0.05 compared with control group, **P<0.01 compared with control group,^ #^P< 0.05compared with L-NAME group ,^ # #^P<0.01 compared with L-NAME.

In order to find out the dynamic changes of NO in different tissues after PM2.5 exposure, the NO in lung, blood, and vessel were tested at 0, 0.5, 1, 6, and 24hrs after the single exposure to PM2.5. The results revealed that the NO in alveolar lavage fluid and lung tissue in PM2.5 group were significantly higher than that of control group (p<0.05) at 0.5hr, and up to the maximum level at 6hr (p<0.01) (Fig. 4A, B). While in blood, no evident difference for NO was detected between the two groups until 1hr later (Fig. 4C). The NO in blood also reached to maximum level at 6hr (p<0.01) (Fig. 4C). There were no significant changes of NO in vessel (Fig. 4D). In NOS inhibitor (L-NAME) treated group , NO levels in alveolar lavage fluid, lung tissue and blood in PM2.5 group were significantly reduced in comparision with those of the exposure group at all time points (Fig. 4A-C).

### The dynamic effect on NOS after single exposure to PM2.5

**Figure 5:**
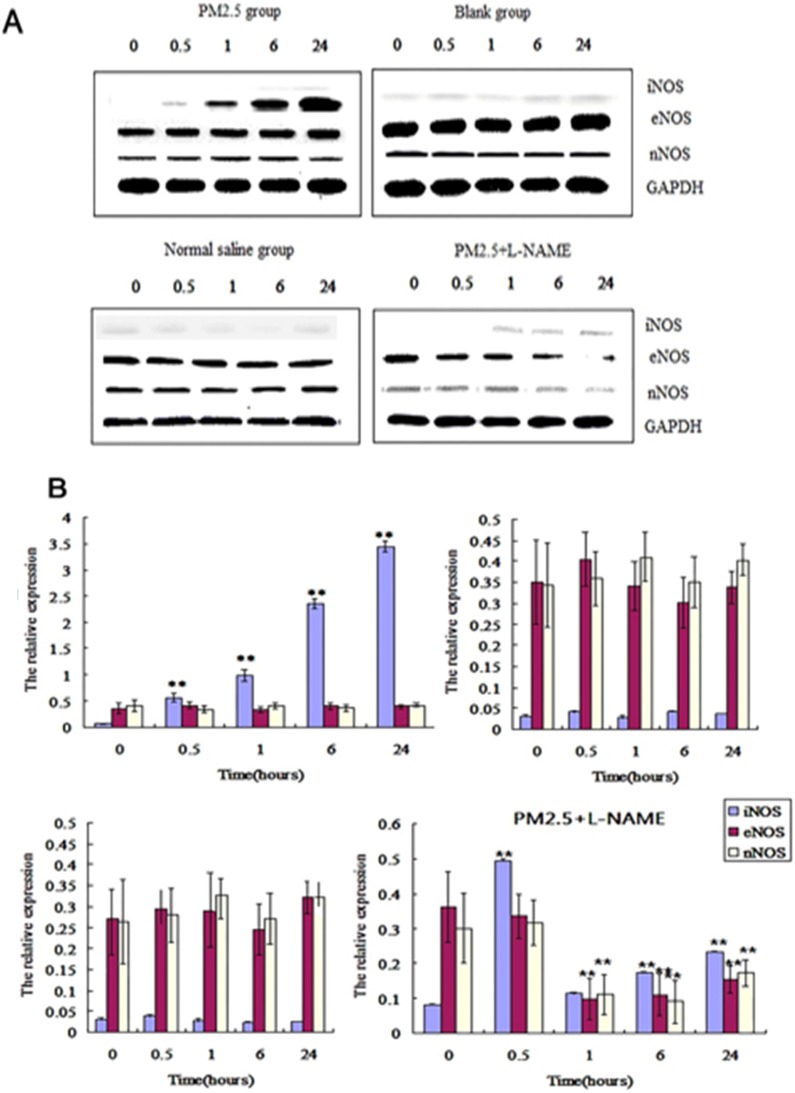
** The dynamic effects of single exposure to PM2.5 on NOSs in vessel. (A)** Western blot analysis for the protein level of NOSs in vessel (n=4). **(B)**The qPCR assay showed the transcriptional level of NOSs in vessel (n=10).*P<0.05 compared with zero hour, **P< 0.01 and compared with the zero hour.

Western-blotting showed that iNOS in vessel was significantly increased at 0.5, 1, 6, 24hrs after exposures to PM2.5 than that of before exposure, while eNOS and nNOS were not significantly changed at all time points (Fig. 5A). qPCR detection also showed that the mRNA transcription of iNOS significantly raised at 0.5hr after exposure to PM2.5, and reached to its maximum at 6hr, while no significant changes occurred in eNOS and nNOS mRNA transcriptions (Fig. 5B). The content of iNOS were significantly increased in vessel at 0.5, 1, 6, 24hrs, but neither eNOS nor nNOS significantly changed in any time points (Fig. 5).

### The dynamic effect on oxidative stress in lung, blood and vessel after single exposure to PM2.5

**Figure 6:**
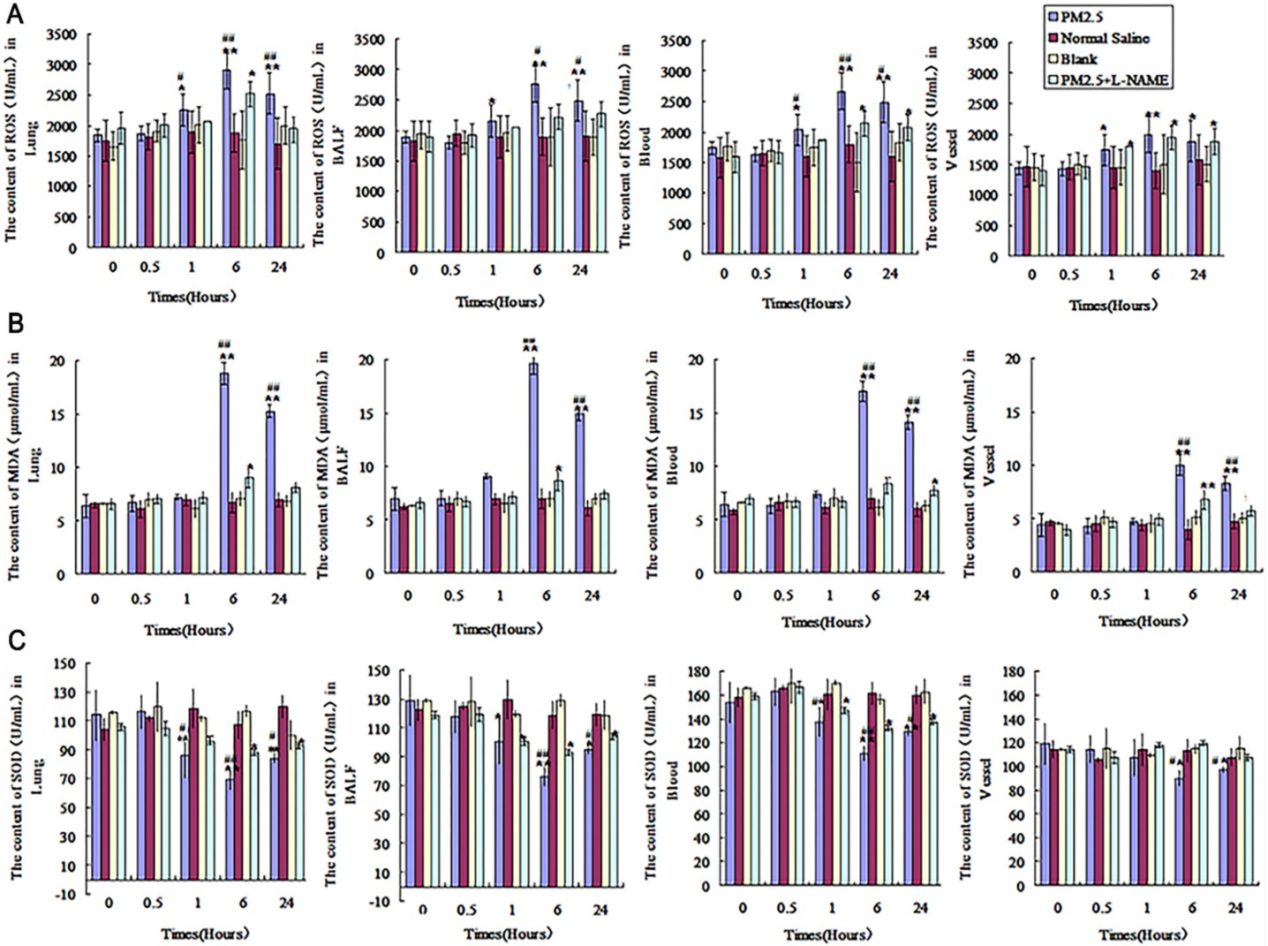
**The dynamic effects of single exposure to PM2.5 on oxidative stress in various tissues.** Oxidative stress indicators were detected in lung, alveolar lavage fluid ,blood and vessel at 0, 0.5, 1, 6 and 24hrs. (A)The ROS levels (n=10). (B) MDA levels (n=10). (C) SOD levels (n=10). *P<0.05 compared with control group,**P<0.01 compared with control group, ^#^P<0.05 compared with L-NAME group, ^# #^P<0.01 compared with L-NAME group.

To understand the dynamic effects of PM2.5 exposure on oxidative stress, SOD, NO, ROS and methylenedioxyamphetamine (MDA) were detected in lung, blood and vessel at 0, 0.5, 1, 6 and 24hrs. It was found that there were no significant changes for ROS and SOD at 0.5hr compared with that in control group (Fig. 6A and C). The levels of ROS in alveolar lavage fluid, lung tissue and blood were significantly elevated at 1hr, and up to the maximum value at 6hr (p<0.01) (Fig. 6A). However, SOD in alveolar lavage fluid, lung tissue and blood was significantly decreased at 1hr (p<0.05), and down to the lowest at 6hr (Fig. 6C). ROS and SOD in vessel changed significantly after exposure for 6hr compared with that in control group (p<0.05) (Fig.6A and C). MDA was increased in alveolar lavage fluid, lung tissue, blood and vessel after exposure to PM2.5 for 6hr (p<0.01), and reach to the maximum level at 24hr (Fig.6B). L-NAME could effectively decrease oxidative stress induced by exposure to PM2.5, the changes of MDA, ROS and SOD in alveolar lavage fluid, lung tissue, blood and vessel were weakened to different degree (Fig. 6).

### The influence of single exposure to PM2.5 on the level of TNF-α and IL-6 in lung tissue, blood and vessel

**Figure 7:**
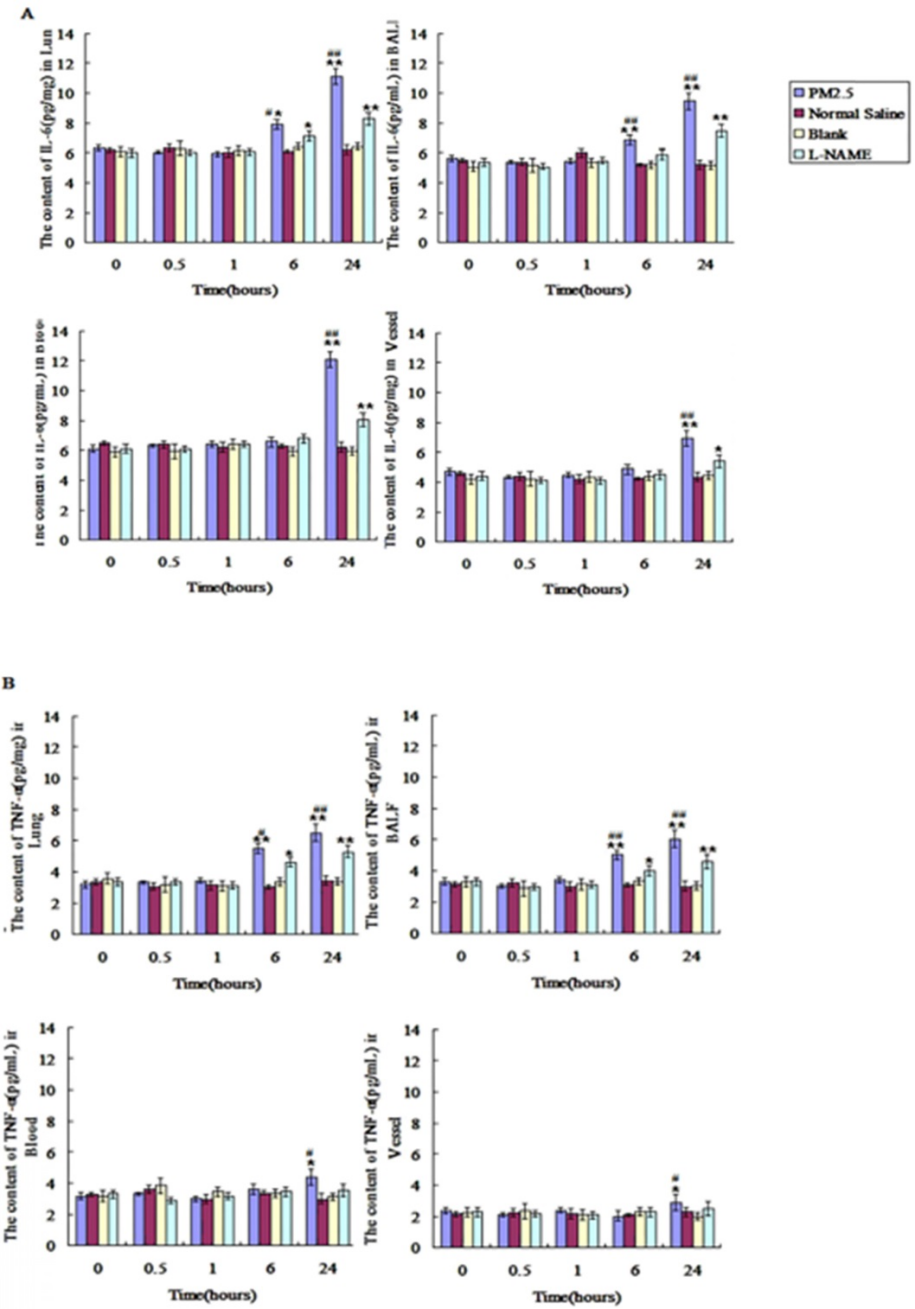
** The dynamic changes of inflammatory factors in lung, BALF, blood and vessel after single PM2.5 exposure.** The level of IL6 and TNF-α in lung, BALF, blood and vessel at 0, 0.5, 1, 6 and 24hrs after single PM2.5 exposure. **(A)** IL-6(n=10). **(B)**TNF-α(n=10). *P<0.05 compared with control group, **P<0.01 compared with control group, ^#^P<0.05 compared with L-NAME group,^ # #^P<0.01 compared with L-NAME.

Compared with control group, TNF-α and IL-6 were significantly increased at the time point of 6hr after PM2.5 exposure in lung tissue and alveolar lavage fluid, and both of them were increased to the peak at 24hr (p < 0.01). No significant differences of TNF-α and IL-6 between control and exposure group were found in the blood and vessel at 6 hours after PM2.5 exposure, while a marked increase in blood and vessel at 24hr after PM2.5 treatment (Fig.7A and B). Compared with the exposure group, L-NAME inhibited the increase of IL-6 and TNF-a induced by PM2.5 exposure in lung, blood and vessel, and IL-6 and TNF-α in L-NAME group were decreased significantly after exposure for 24hr (Fig. 7A and B).

### PM2.5 exposure may activate iNOS via reducing its DNA methylation

**Figure 8:**
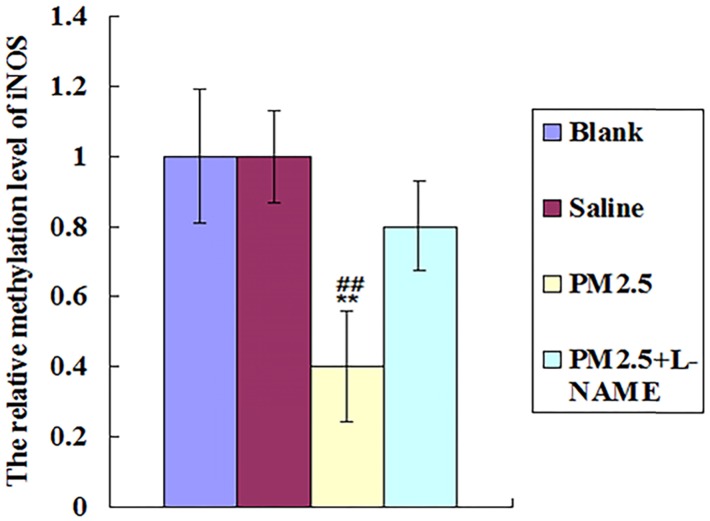
** The effects of multiple exposure to PM2.5 on the methylation level of the iNOS.** The relative methylation level of iNOS was detected in vessel (n=10).*P<0.05 compared with control group,**P<0.01 compared with control group, #P<0.05 compared with L-NAME group, # # P<0.01 compared with L-NAME.

PM2.5 collected from Oct. to Dec. 2015 in Beijing city was analysed, and PM2.5- adsorbed metal elements and polycyclic aromatic hydrocarbons (PAH) were shown in Table 3. Among these components of the PM2.5 in our study, Zn, Ni, Al, Pb and PAHs were all reported to activate iNOS directly^28^-^32^, suggesting the direct activation of iNOS by these PM2.5 components. Especially, PAHs were verified to upregulate iNOS expression via the decrease of their DNA methylation level 33-35. Indeed, our results showed that PM2.5 exposure could reduce the DNA methylation of iNOS at 0.5hr, highly supporting the potential role of PAHs in activating iNOS (Fig. 8).

### The Composition and Content of PM2.5

The component and content of PM2.5 collected at different areas and periods are different. The data showed the composition and content of PM2.5 collected between the 2nd and 3rd ring road from Oct. to Dec. 2015 in Beijing China (Table 3).

## Discussion

PM2.5 exposure has been determined to be an etiological factor of cardiovascular diseases by American Heart Association. It is reported that PM2.5 exposure induces vascular dysfunction, showing a positive correlation with the morbidity of cardiovascular diseases, in which oxidative stress and inflammation induced by PM2.5 play important roles, but the mechanism has not yet been elucidated in detail.

First, our study showed that PM2.5 exposure exerted significant influences on the development of vasculopathy via oxidative stress and inflammation, as well as its negative effects on vascular function, in which NO may play an important role. The multiple exposures to PM2.5 remarkably enhanced the inflammatory factor levels in both blood and vessel, including IL-6, TNF-α and ICAM-1. In addition, PM2.5 also significantly upregulated the MDA level, while downregulated the SOD activity. Accompanied with these pathological changes, it was further found that TIMP-1 was decreased while MMP9 was increased in vessel after the multiple exposure to PM2.5, suggesting a significant adverse remodeling of vessel structure. We further demonstrated that PM2.5-induced decrease of the aortic ring intension caused by PE stimulation was completely recovered after ODQ treatment, illustrating that the abnormality of the NO/SGC signal occurred when exposed to PM2.5. Both Ca^2+^ ionophore A23187 and SNP induced vessel diastole were also significantly decreased after PM2.5 exposure. All these results strongly suggested that PM2.5 could influence the release of NO to enhance vasoconstriction.

Actually, NO is widely involved in oxidative stress and inflammation, as well as vasculopathy in body. Many studies have demonstrated that NO is closely related to both oxidative stress and inflammation induced by PM2.5 exposure in respiratory system. Frank d. Gilliland found that short-term PM2.5 exposure could upregulate the quantity of asthma children's FeNO, accompanied with increased oxidative stress and inflammation in respiratory system 36. In addition, it is also reported that PM2.5 exposure increases the amount of exhaled NO in the people suffered from the respiratory illnesses, and the exhaled NO positively correlates with both the PM2.5 exposure level and the incidence of respiratory system diseases 37-40. We detected a significant upregulation of iNOS, but not cNOS in vessel, suggesting a pathological change via the abnormally elevated NO generated by iNOS. As a result, we hypothesized that NO might play a central role in the development of vasculopathy after PM2.5 exposure.

In addition, our study further demonstrated that iNOS-induced NO in lung was the initiation factor of vascular dysfunction after PM2.5 exposure. In order to explore the detailed mechanism of NO to aggravate vascular dysfunction, we adopted the single PM2.5 exposure model of ApoE-/- mice to avoid the interference caused by confounding factors of multiple PM2.5 exposure. Our results indicated that NO was increased significantly at 0.5hr after exposure to PM2.5, meanwhile, ROS, SOD and MDA significantly changed at 1hr later, and the significant change of IL-6 and TNF-α only happened at 6hr in lung. Additionally, NO and oxidative stress indicators were not obviously changed until 1hr in blood, and both IL-6 and TNF-a obviously changed until 24hr. These results suggested that NO could be elevated abnormally after exposure to PM2.5 at first, then caused lung oxidative stress and inflammation, and the inflammation lagged behind oxidative stress after PM2.5 exposure. Not only that, we confirmed that the great change of NO appeared before ROS, suggesting that NO was a key initial factor for oxidative stress and inflammation induced by PM2.5 exposure. Indeed, NOS inhibitors could obviously reduce NO after PM2.5 exposure in lungs and blood, and also suppressed subsequent oxidative stress and inflammation in lung. iNOS was significantly increased in PM2.5 exposure group, whereas eNOS and nNOS were not obviously changed, indicating that iNOS was the key enzyme for NO abnormal elevation. All these results indicated that PM2.5 exposure activated iNOS in lung to produce abnormally elevated NO to induce oxidative stress, then led to inflammation in lung, finally caused systemic upregulation of NO, as well as the following systemic oxidative stress and inflammation successively, causing the aggravation of vascular dysfunction. Consistent with our research is that the excessive generation of NO has been demonstrated to cause nitrification reaction and oxidative stress in lung, which jointly controls inflammation in respiratory tract caused by PM2.5 exposure [41.42].

At last, our study showed that the PM2.5 exposure directly activated iNOS in lung to produce excess NO as the initiation factor of vascular dysfunction. It is well known that the toxic effects of PM2.5 are closely related with its composition. The component analysis indicated that the PM2.5 collected from Beijing in our study contained large amounts of Zn, Cu, Ni, Al, Pb and PAHs, all of which are reported to activate iNOS via their direct interaction 27-31. Especially, PAHs is verified to upregulate iNOS expression via decreasing its DNA methylation level 33-35. In line with these studies, our results indicated that PM2.5 exposure reduced the DNA methylation of iNOS in 0.5hr, highly supporting the potential role of PAHs in activating iNOS. Although these specific components as potential iNOS agonists were common ingredients of PM2.5 from various independent studies, whether the bio-effects of PM2.5 from other areas or other periods of time display similar results still need to be further studied.

In summary, we demonstrated for the first time that iNOS was the key inducer for PM2.5 exposure-activated oxidative stress and inflammation, and confirmed that NO was a critical initiating factor for vascular dysfunction after exposure to PM2.5 collected from Beijing. Our study lays a theoretical foundation for the potential novel treatment strategies against cardiovascular diseases aggravated by exposure to PM2.5.

## Figures and Tables

**Table 1:** The Primers for methylation detection

Gene	Primer	
iNOS	Forward	5'-TTGTTTGTATTTAGTAAGTTGTTTTTT-3′
	Reverse	5'-AAAATCCTATAATTCTCCCCTCTTCT-3′
iNOS M	Forward	5'-TTGTTTGTATTTAGTAAGTTGTTTTTT-3′
	Reverse	5'-AAAATCCTATAATTCTCCCCTCTTCT-3′
iNOS U	Forward	5'-ATCATTACCCAATTTAATAAACAACG-3′
	Reverse	5'-TTATGTTAGGATTATGGTTTTGAATG-3′

**Table 2:** The Primers for qPCR

Gene	Primer	
TNFα	Forward	5'-GTCTCAGCCTCTTCTCATTCCTG-3′
	Reverse	5'-TCCTCCACTTGGTGGTTTGCTAC-3′
IL-6	Forward	5'-AATGGCAATTCTGATTGTATGAAC-3′
	Reverse	5'-ACTCCTTCTGTGACTCCAGCTTAT-3′
GAPDH	Forward	5'-TTGTCAAGCTCATTTCCTGGTATG-3′
	Reverse	5'-GCCATGTAGGCCATGAGGTC-3′
MMP9	Forward	5'-GCTTAGATCATTCCAGCGTGCC-3′
	Reverse	5'-AGGTATAGTGGGACACATAGTGG-3′
TIMP1	Forward	5'-CGAGACCACCTTATACCAGCG-3′
	Reverse	5'-GCGGTACCGGATATCTGCG-3′
iNOS	Forward	5'-GAATCTTGGAGCGAGTTGTGG-3′
	Reverse	5'-AGGAAGTAGGTGAGGGCTTGG-3′
eNOS	Forward	5'-GACCCTCACCGCTACAACAT-3′
	Reverse	5'-GCCTTCTGCTCATTTTCCAG-3′
nNOS	Forward	5'-GCTTCAGGAATATGAGGAATGG-3′
	Reverse	5'-TGATGGAATAGTAGCGAGGTTGT-3′

**Table 3:** Composition and Content of PM2.5

Pollutant	concentration
PM_2.5_	70.7µg/m^3^
NAP	0.84ng/m^3^
FLU	7.65ng/m^3^
ANA	6.55ng/m^3^
PHE	2.9ng/m^3^
FLT	4.63ng/m^3^
PYR	7.41ng/m^3^
CHR	6.59ng/m^3^
BaA	7.7ng/m^3^
BbF	7.43ng/m^3^
BkF	6.94ng/m^3^
BaP	9.15ng/m^3^
DBA	7.29ng/m^3^
BPE	6.53ng/m^3^
∑PAHs	81.61ng/m^3^
Be	0.1ng/m^3^
Al	143.23ng/m^3^
Ti	3.59ng/m^3^
Cr	4ng/m^3^
Co	0.61ng/m^3^
Ni	18.44ng/m^3^
Cu	53.78ng/m^3^
Zn	1107.89ng/m^3^
As	19.81ng/m^3^
Se	11.08ng/m^3^
Mo	4.53ng/m^3^
Ag	0.39ng/m^3^
Cd	4.46ng/m^3^
Sn	8.99ng/m^3^
Sb	10.53ng/m^3^
Ba	19.16ng/m^3^
Tl	1.9ng/m^3^
Pb	237.73ng/m^3^
